# Supplementary material for: Inhibition mechanism of the chloride channel TMEM16A by the pore blocker 1PBC
Source: Nat Commun. 2022 May 19;13:2798. doi: 10.1038/s41467-022-30479-1 (PMC9120017; doi:10.1038/s41467-022-30479-1)
Supplement: Supplementary file 1 — Supplementary Information [file 41467_2022_30479_MOESM1_ESM.pdf]

Supplementary Information

Inhibition mechanism of the chloride channel TMEM16A by the pore blocker 1PBC

Andy K. M. Lam, Sonja Rutz and Raimund Dutzler

Supplementary Figures

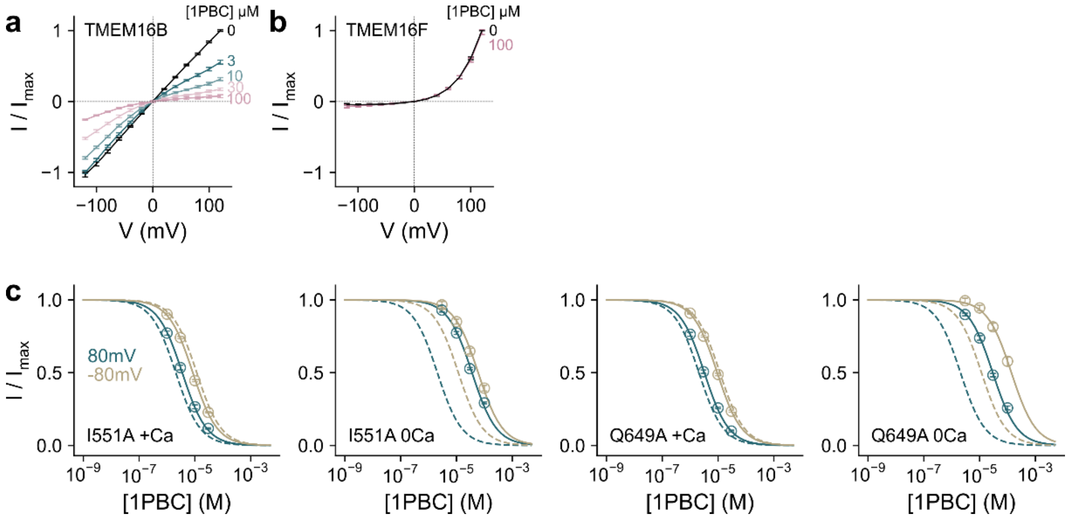

Supplementary Fig. 1: Effect of 1PBC on TMEM16B, TMEM16F, and selected constitutively active mutants of TMEM16A. a, b, Steady-state current-voltage relationship of mouse TMEM16B (**a**) and TMEM16F (**b**) at the indicated concentrations of 1PBC applied to the intracellular side of the membrane at a saturating Ca^{2+} concentration (15 µM and 300 µM respectively). Data are averages of 5 and 6 biological replicates respectively, errors are SEM. **c,** 1PBC concentration-response relations of the indicated constitutively active mutants of TMEM16A measured either at a saturating Ca^{2+} concentration (2 µM) or at zero Ca^{2+} at -80 and 80 mV. Data are averages of 6, 9, 7, and 7 biological replicates respectively, errors are SEM. Solid lines are fits to the Hill equation. Dashed lines show the relations of WT.

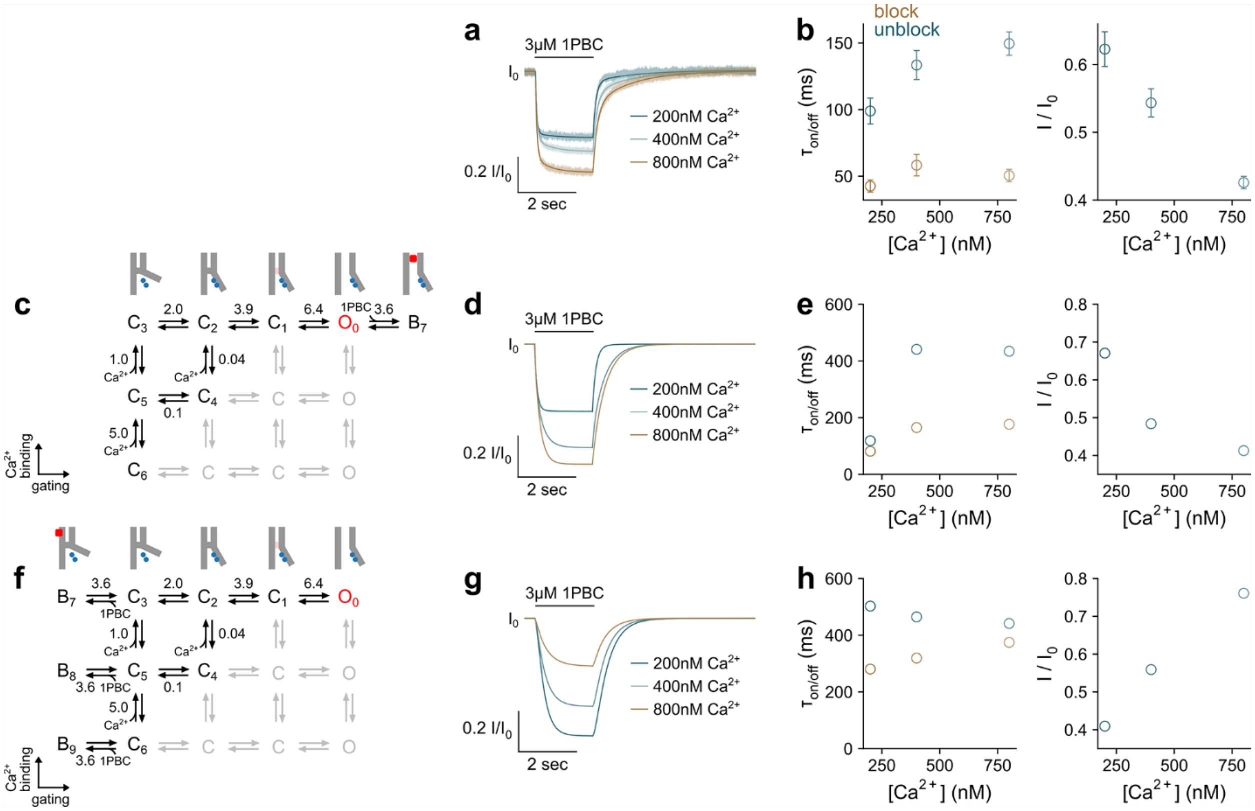

Supplementary Fig. 2: State dependence of 1PBC block. a, Kinetics of 1PBC block at an intermediate 1PBC concentration and the indicated sub-saturating Ca^{2+} concentrations in ultra-fast concentration-jump experiments at +80 mV in the inside-out configuration. 1PBC was applied from the intracellular side. The current traces were corrected for rundown using a linearly decaying baseline, and were normalized to the respective steady-state currents in the absence of 1PBC (I/I_0). Solid lines are empirical fits to a sum of two exponentials. **b,** Time constants and fractional blockade by 1PBC. Data were obtained via an empirical exponential fit and are averages of 15 biological replicates, errors are SEM. **c,** Open-state block model. **d,** Time course of 1PBC block calculated using the open-state block model shown in (**c**) at the indicated Ca^{2+} concentrations. **e,** Time constants of blocking and unblocking and fractional inhibition empirically determined from the calculated time course (**d**) via a fit to a sum of two exponentials. **f,** Closed-state antagonism model. **g,** Time course of 1PBC inhibition calculated using the closed-state antagonism model shown in (**f**) at the indicated Ca^{2+} concentrations. **h,** Time constants of blocking and unblocking and fractional inhibition empirically determined from the calculated time course (**g**) via a fit to a sum of two exponentials. **b, e, h,** The fast time constant of the two exponentials is plotted. **c, f,** K_d in µM and forward equilibrium constants are shown. For the calculations, the gating parameters were as in our previous study[28] and the values of the blocking parameters were: K_d of 1PBC = 3.6 µM (as determined in Fig. 2a, b) and k_{on} = 1 x 10^6 $M^{-1}s^{-1}$. The same values

were used for the two models to allow a direct comparison. Grey, pore helices; pink, destabilized gate; blue spheres, Ca^{2+} ions; red, inhibitor. **c-h,** The time course of 1PBC block at different Ca^{2+} concentrations was calculated using Eqs. 4-13. A qualitative agreement between the data and the open-state block model is observed, where increasing Ca^{2+} concentrations slow unblocking and promote steady-state blockade. In contrast, a closed-state antagonism model predicts that increasing Ca^{2+} concentrations would antagonize inhibition by 1PBC, likely due to a corresponding depletion of the closed states.

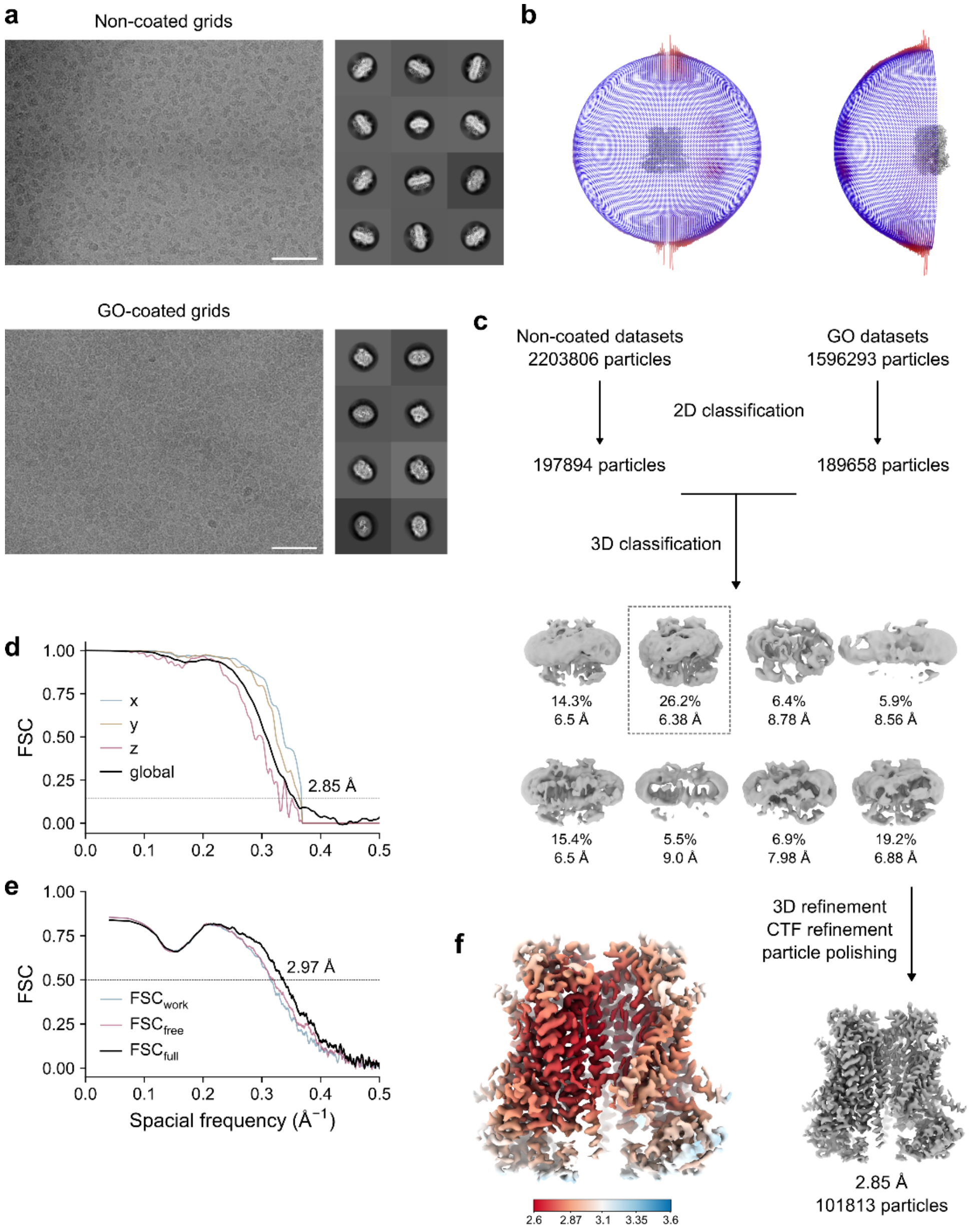

Supplementary Fig. 3: Cryo-EM reconstruction of 1PBC/Ca^{2+}-bound TMEM16A. a, Representative micrographs (scale bar: 50 nm) and 2D class averages of TMEM16A in the presence of 1PBC and Ca^{2+} obtained from (top) non-coated and (bottom) GO-coated Quantifoil grids. **b,** Angular distribution of particle projections used in the final refinement. **c,** Data processing workflow. **d,** Global and directional Fourier shell correlations (FSCs) between the half-maps estimated using the 3DFSC server (https://3dfsc.salk.edu/)[64]. **e,** Model-versus-map FSCs. The curves correspond to the FSC between the final model and the summed half-maps (FSC_{full}), half-map 1 and the shaken final model refined against half-map 1 (FSC_{work}), and half-map 2 and the shaken refined model (FSC_{free}). **f,** Local resolution of the final map estimated using RELION.

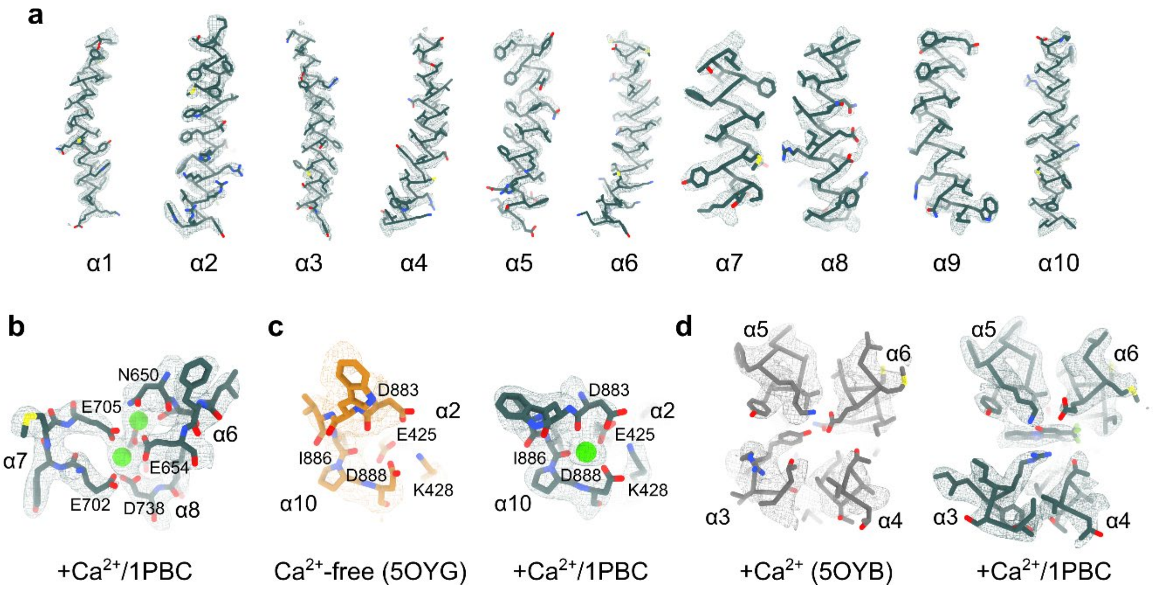

Supplementary Fig. 4: Cryo-EM densities. a, Sections of cryo-EM densities of the transmembrane region superimposed on the refined model. **b-d,** Sections of cryo-EM densities at (**b**) the canonical Ca^{2+} binding site, (**c**) the additional third Ca^{2+} binding site near the dimer interface, and (**d**) the 1PBC binding site superimposed on the indicated structures. The Ca^{2+}-bound WT structure (5OYB) shows the rebuilt model with a 'down' conformation of α3.

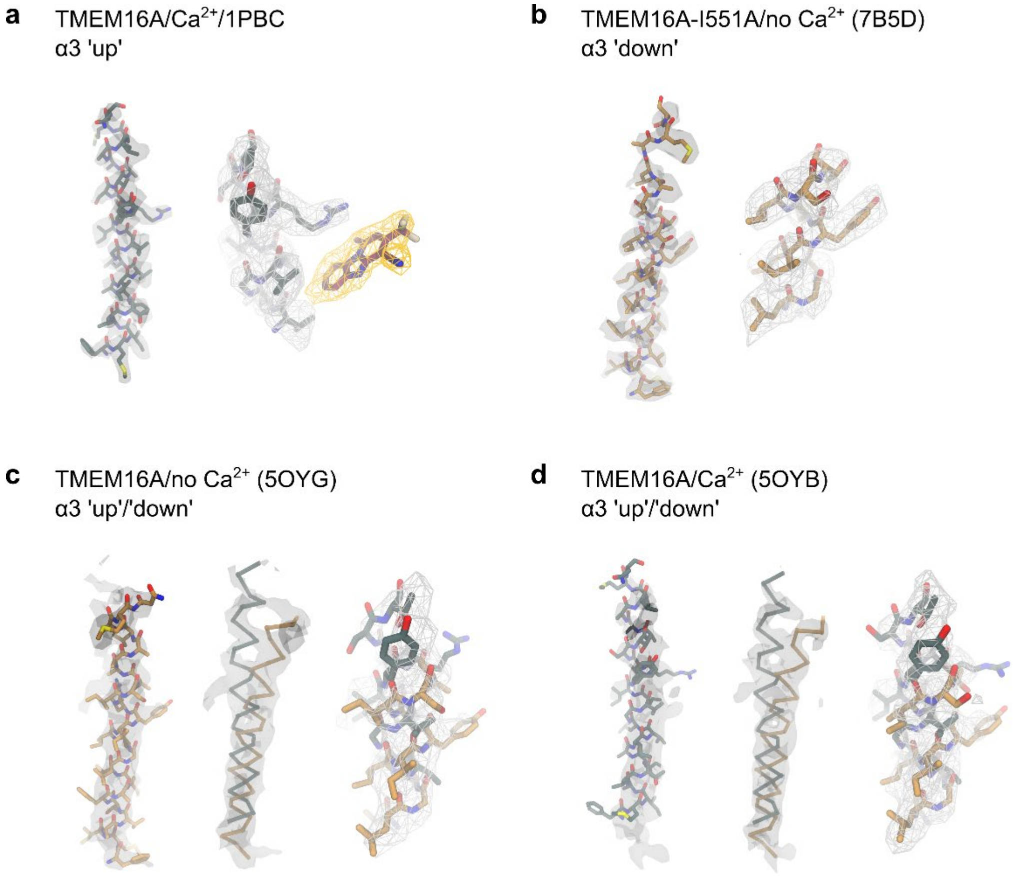

Supplementary Fig. 5: α3 conformations. a-d, Sections of cryo-EM densities of the helix α3 superimposed on the indicated structures. The 'up' conformation is shown in green and the 'down' arrangement in gold. **c, d,** α3 of the Ca^{2+}-free and Ca^{2+}-bound states was remodeled in light of the well-defined conformations of this helix in the here obtained 1PBC/Ca^{2+}-bound structure and that of the constitutively active mutant I551A in the absence of Ca^{2+}. **b-d,** The PDB ID's of the original entries are indicated.

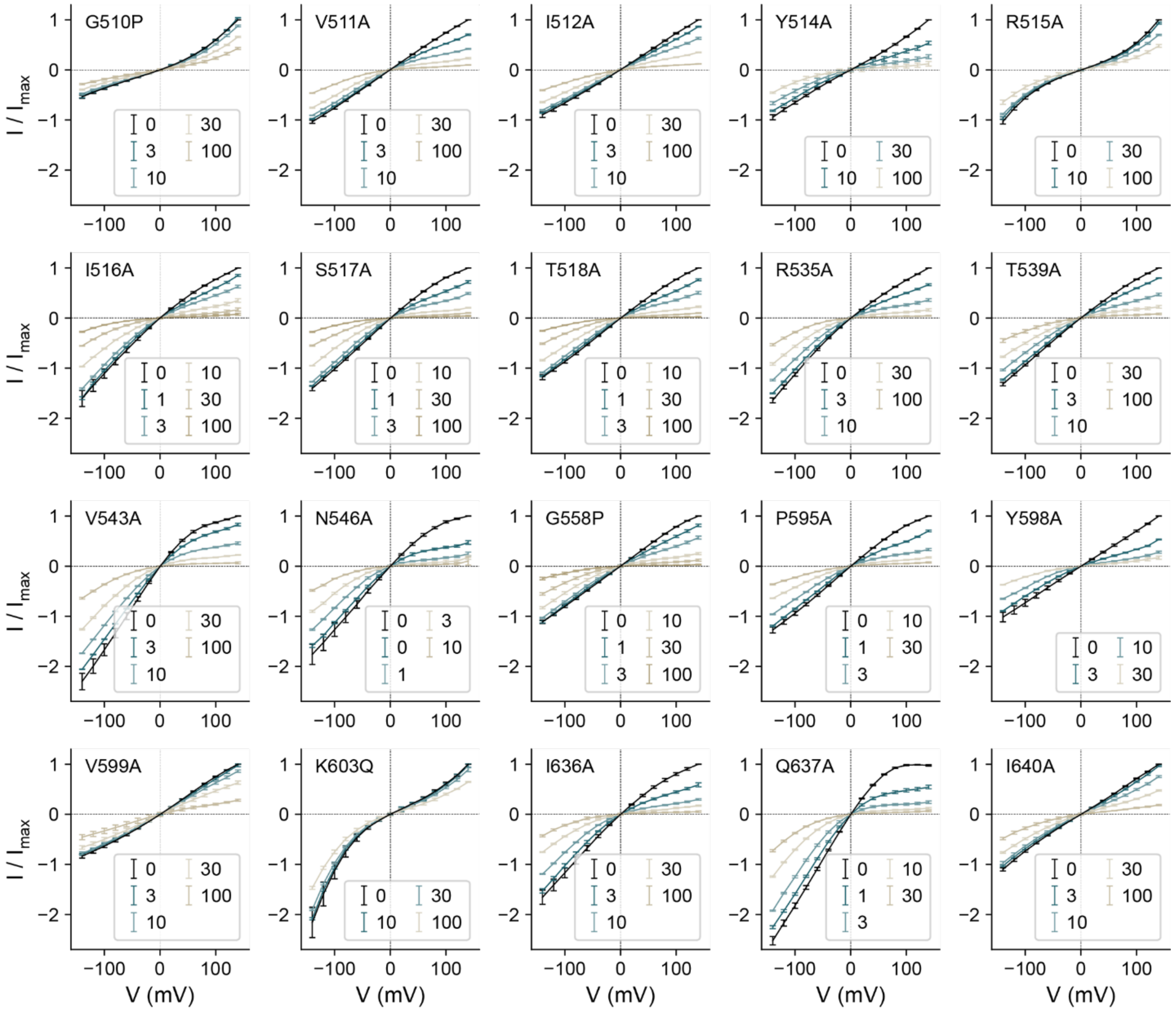

Supplementary Fig. 6: Current-voltage relations of mutants. Steady-state current-voltage relations of mutants at the indicated concentrations of 1PBC (in µM) applied to the intracellular side of the membrane in the presence of a saturating Ca^{2+} concentration as indicated in Supplementary Table 1. Data are averages of the indicated number of biological replicates shown in Supplementary Table 1, errors are SEM.

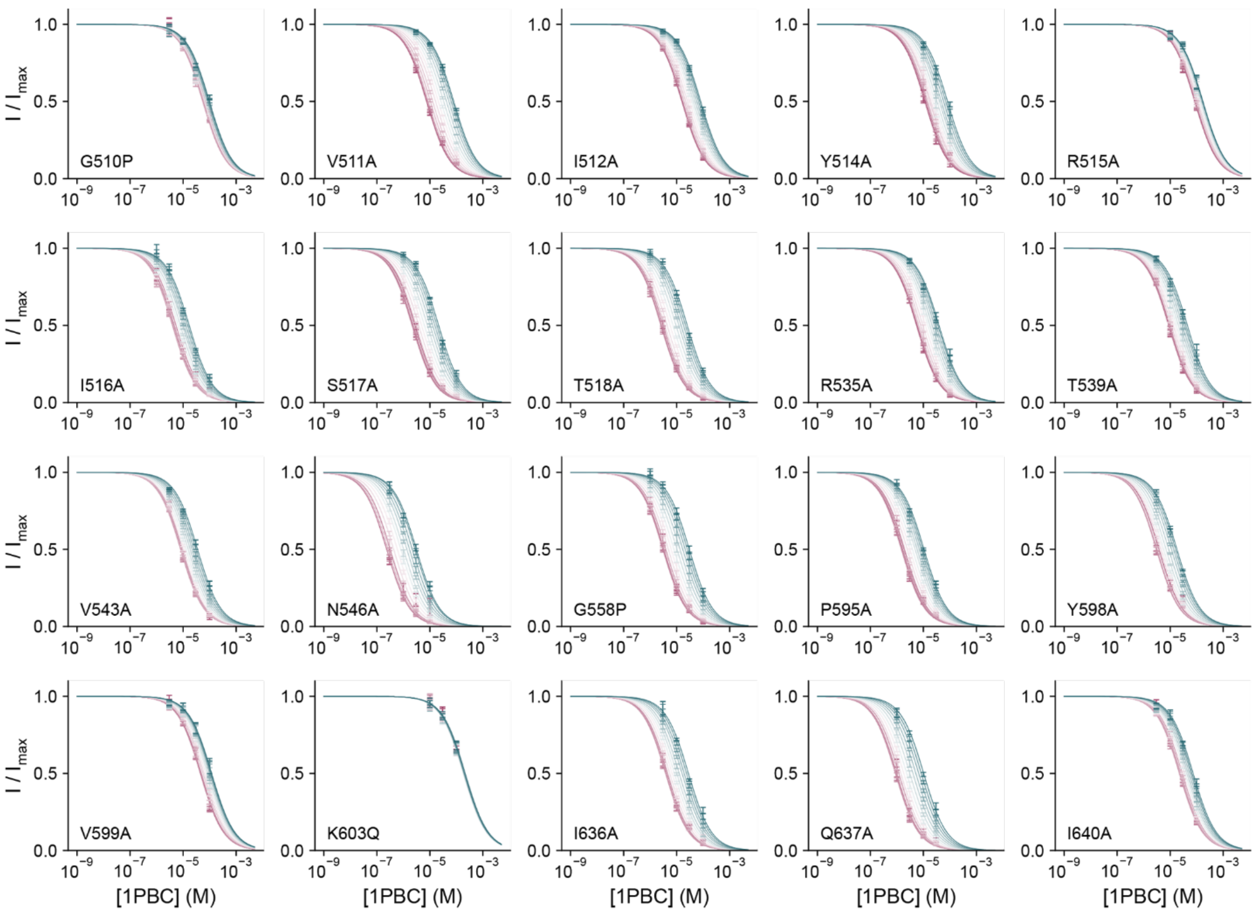

Supplementary Fig. 7: Concentration-response relations of mutants. 1PBC concentration-response relations of mutants in the presence of a saturating Ca^{2+} concentration at voltages from -140 (green) and 140 mV (red), ΔV = 20 mV. Data are averages of the indicated number of biological replicates shown in Supplementary Table 1, errors are SEM. Solid lines are fits to the Hill equation.

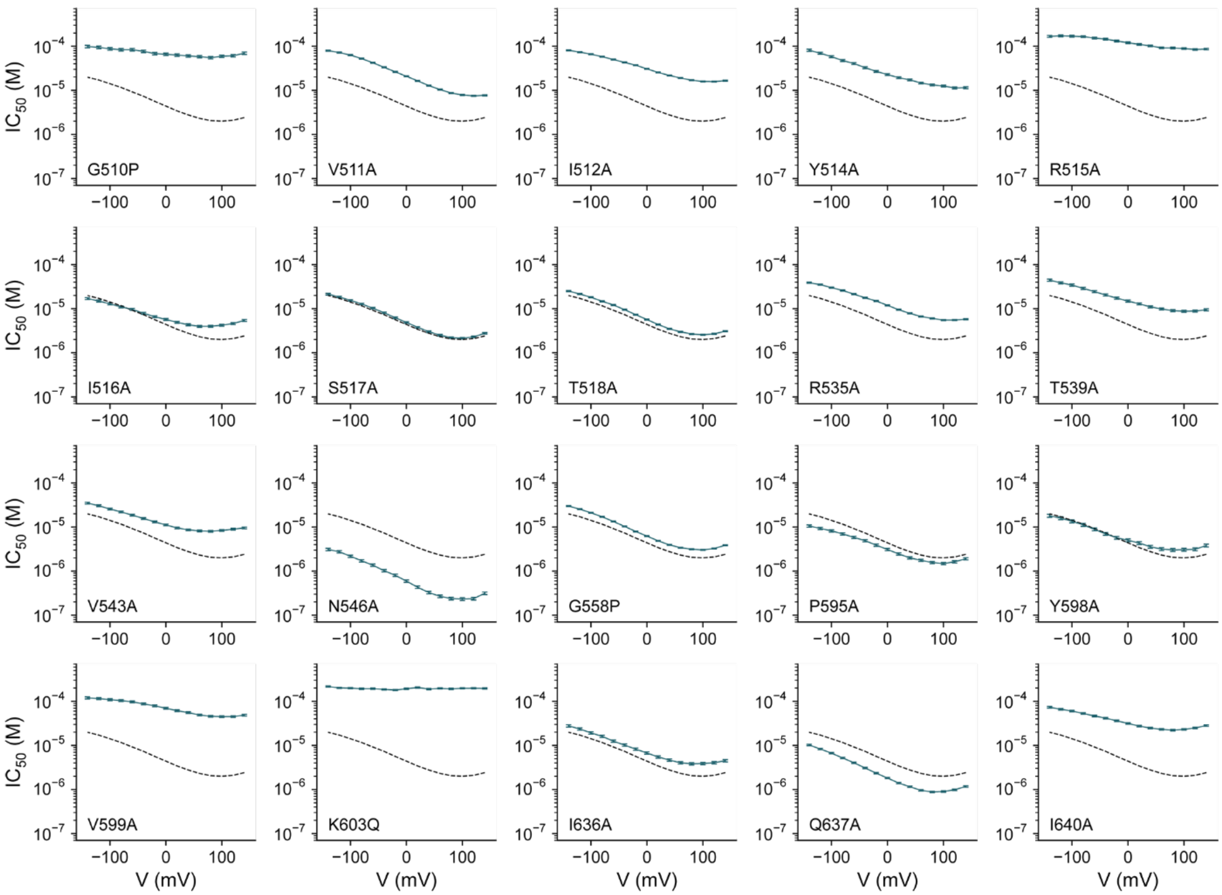

Supplementary Fig. 8: Voltage dependence of 1PBC block of mutants. IC_{50} values of mutants obtained in Supplementary Fig. 7 at the indicated voltages. Data are best-fit values of the indicated number of biological replicates shown in Supplementary Table 1, errors are 95% CI. Dashed lines are the relation of WT.

a

$$\begin{array}{ccccccccc}
C_3^{x_2} & \leftrightarrow & C_2^{x_2} & \leftrightarrow & C_1^{x_2} & \leftrightarrow & O_0^{x_2} & \leftrightarrow & B_7^{x_2} \\
\updownarrow & & \updownarrow & & & & & & \\
C_5^{x} & \leftrightarrow & C_4^{x} & & & & & & \\
\updownarrow & & & & & & & & \\
C_6 & & & & & & & &
\end{array}$$

b

$$\begin{array}{ccccccccc}
B_7^{x_2} & \leftrightarrow & C_3^{x_2} & \leftrightarrow & C_2^{x_2} & \leftrightarrow & C_1^{x_2} & \leftrightarrow & O_0^{x_2} \\
 & & \updownarrow & & \updownarrow & & & & \\
B_8^{x} & \leftrightarrow & C_5^{x} & \leftrightarrow & C_4^{x} & & & & \\
 & & \updownarrow & & & & & & \\
B_9 & \leftrightarrow & C_6 & & & & & &
\end{array}$$

Supplementary Fig. 9: Mechanistic schemes. a, Open-state block. The scheme depicts a previously described gating mechanism[28] with addition of a blocker binding step to the open state. **b,** Closed-state antagonism where blocker binding steps were added to the states C_3, C_5, and C_6. **a, b,** C, O, and B correspond to the closed, open, and blocked/inhibited states respectively, and the subscripts denote the number assigned to the states.

Supplementary Tables

Supplementary Table 1: IC_{50} of 1PBC of mutants.

	[Ca^{2+}]	n	140	120	100	80	60	40	20	0	-20	-40	-60	-80	-100	-120	-140
**WT**	2	6	2.41	2.1	2.0	2.06	2.32	2.77	3.42	4.4	5.63	7.34	9.2	11.6	14.1	17.2	19.9
**G510P**	15	9	69.5	60.9	59.3	54.9	58.2	60.2	63.2	65.7	68.2	76.6	83.4	83.4	87.4	94.4	98.6
**V511A**	2	7	7.71	7.49	7.86	8.67	10.4	12.8	16.4	20.8	26.3	33.3	42.3	52.4	62.9	72.1	79.5
**I512A**	2	5	16.6	15.8	15.9	17.0	19.0	21.8	25.5	30.7	37.1	42.9	50.1	57.9	66.1	73.6	80.9
**Y514A**	2	5	11.5	11.3	12.5	13.3	14.6	17.3	19.4	22.8	26.9	32.7	40.6	47.4	58.0	69.6	81.0
**R515A**	4	8	86.1	84.4	88.7	91.4	92.1	102.0	110.0	120.0	131.0	145.0	153.0	166.0	169.0	172.0	168.0
**I516A**	2	5	5.41	4.6	4.22	4.0	3.97	4.32	4.91	5.69	6.57	7.87	9.65	11.0	12.9	15.0	17.0
**S517A**	2	5	2.76	2.29	2.14	2.2	2.51	3.01	3.78	4.87	6.24	8.08	10.3	12.8	15.5	18.4	21.4
**T518A**	2	6	3.08	2.69	2.55	2.66	2.99	3.5	4.37	5.67	7.32	9.5	12.2	14.7	18.1	21.5	25.1
**R535A**	2	7	5.76	5.53	5.51	6.02	6.65	7.82	9.48	11.9	14.9	17.7	21.4	25.9	30.1	35.1	38.8
**T539A**	2	5	9.45	8.83	8.74	9.02	9.86	11.0	12.9	14.9	17.3	20.5	24.4	28.9	34.3	38.6	44.4
**V543A**	15	7	9.55	8.93	8.37	8.02	8.16	8.65	9.55	11.2	13.1	15.6	18.6	22.0	25.7	30.4	34.9
**N546A**	2	6	0.315	0.238	0.233	0.239	0.269	0.326	0.431	0.594	0.805	1.02	1.36	1.72	2.17	2.75	3.1
**G558P**	15	8	3.86	3.26	3.03	3.14	3.4	3.94	4.87	6.23	7.94	10.4	13.4	17.1	21.1	25.4	30.0
**P595A**	2	5	1.91	1.63	1.48	1.57	1.75	2.0	2.46	3.1	3.88	4.91	5.83	7.0	8.19	9.33	10.5
**Y598A**	2	5	3.81	3.13	3.06	3.04	3.16	3.57	4.35	5.0	5.71	7.02	8.86	11.2	13.5	15.9	18.0
**V599A**	15	7	48.5	45.1	45.0	45.8	48.9	55.5	61.3	69.5	79.2	87.5	97.2	104.0	109.0	115.0	119.0
**K603Q**	4	8	196.0	198.0	197.0	192.0	196.0	190.0	206.0	193.0	180.0	187.0	193.0	193.0	199.0	202.0	216.0
**I636A**	15	4	4.5	4.05	3.89	3.83	4.03	4.65	5.47	6.73	8.22	10.2	12.5	16.0	19.2	23.6	27.8
**Q637A**	2	7	1.17	0.985	0.898	0.88	0.956	1.15	1.4	1.82	2.34	3.08	4.04	5.19	6.66	8.29	10.2
**I640A**	15	5	28.2	24.9	23.1	22.1	23.1	24.7	27.5	31.5	36.1	41.5	46.6	53.0	60.2	66.0	73.5

IC_{50} in µM; [Ca^{2+}], intracellular Ca^{2+} concentration in µM; n, number of biological replicates; header, applied voltage in mV.

Supplementary Table 2: 95% CI of IC_{50} of mutants.

	[Ca^{2+}]	n	140	120	100	80	60	40	20	0	-20	-40	-60	-80	-100	-120	-140
**WT**	2	6	0.071	0.062	0.059	0.061	0.068	0.081	0.1	0.13	0.16	0.21	0.27	0.34	0.42	0.51	0.59
**G510P**	15	9	4.4	3.8	3.6	3.4	3.6	3.7	3.9	4.1	4.3	4.9	5.4	5.4	5.7	6.3	6.6
**V511A**	2	7	0.2	0.2	0.2	0.23	0.27	0.33	0.42	0.54	0.69	0.88	1.1	1.4	1.8	2.1	2.3
**I512A**	2	5	0.38	0.36	0.36	0.39	0.44	0.5	0.59	0.72	0.88	1.0	1.2	1.4	1.7	1.9	2.1
**Y514A**	2	5	0.54	0.54	0.59	0.62	0.68	0.8	0.89	1.1	1.2	1.5	1.9	2.3	2.9	3.6	4.3
**R515A**	4	8	3.3	3.2	3.4	3.5	3.6	4.0	4.4	5.0	5.6	6.4	6.8	7.6	7.8	8.0	7.7
**I516A**	2	5	0.27	0.23	0.21	0.2	0.2	0.21	0.24	0.28	0.33	0.39	0.48	0.54	0.64	0.75	0.85
**S517A**	2	5	0.1	0.088	0.082	0.084	0.095	0.11	0.14	0.18	0.24	0.31	0.39	0.48	0.59	0.7	0.82
**T518A**	2	6	0.085	0.075	0.071	0.074	0.083	0.097	0.12	0.16	0.2	0.26	0.34	0.41	0.51	0.61	0.71
**R535A**	2	7	0.17	0.16	0.16	0.17	0.19	0.22	0.27	0.34	0.42	0.5	0.61	0.74	0.87	1.0	1.2
**T539A**	2	5	0.49	0.46	0.46	0.47	0.52	0.57	0.67	0.78	0.91	1.1	1.3	1.5	1.9	2.1	2.5
**V543A**	15	7	0.41	0.39	0.36	0.35	0.35	0.37	0.41	0.48	0.56	0.67	0.81	0.96	1.1	1.4	1.6
**N546A**	2	6	0.021	0.016	0.016	0.017	0.018	0.021	0.027	0.036	0.048	0.062	0.082	0.1	0.13	0.17	0.19
**G558P**	15	8	0.088	0.074	0.069	0.072	0.077	0.09	0.11	0.14	0.18	0.24	0.3	0.39	0.48	0.59	0.7
**P595A**	2	5	0.11	0.093	0.085	0.09	0.099	0.11	0.14	0.17	0.22	0.28	0.33	0.4	0.47	0.54	0.61
**Y598A**	2	5	0.28	0.23	0.23	0.23	0.23	0.26	0.31	0.36	0.41	0.5	0.64	0.83	1.0	1.2	1.4
**V599A**	15	7	2.1	2.0	2.0	2.0	2.2	2.5	2.8	3.2	3.7	4.2	4.8	5.2	5.5	5.9	6.2
**K603Q**	4	8	6.2	6.3	6.3	6.1	6.2	6.0	6.7	6.1	5.5	5.8	6.1	6.1	6.4	6.5	7.2
**I636A**	15	4	0.27	0.24	0.23	0.23	0.24	0.27	0.32	0.39	0.47	0.59	0.72	0.94	1.1	1.4	1.7
**Q637A**	2	7	0.042	0.036	0.034	0.033	0.035	0.041	0.049	0.063	0.08	0.11	0.14	0.18	0.24	0.3	0.37
**I640A**	15	5	1.1	0.97	0.89	0.86	0.9	0.96	1.1	1.2	1.4	1.7	1.9	2.2	2.5	2.8	3.2

CI, confidence interval in μM; [Ca^{2+}], intracellular Ca^{2+} concentration in μM; n, number of biological replicates; header, applied voltage in mV.

Supplementary Table 3: List of primers.

Name	F/R	Sequence (5'-to-3')
G510A	F	GTC CTC GCC GTT ATC ATC TAT AGA ATC TCC ACA GCT GCA
G510A	R	GAT AAC GGC GAG GAC GAT TGC AAA TGT CAC TGC GAT CAT
G510P	F	GTC CTC CCC GTT ATC ATC TAT AGA ATC TCC ACA GCT GCA
G510P	R	GAT AAC GGG GAG GAC GAT TGC AAA TGT CAC TGC GAT CAT
V511A	F	CTC GGA GCC ATC ATC TAT AGA ATC TCC ACA GCT GCA GCC
V511A	R	GAT GAT GGC TCC GAG GAC GAT TGC AAA TGT CAC TGC GAT
I512A	F	GGA GTT GCC ATC TAT AGA ATC TCC ACA GCT GCA GCC TTG
I512A	R	ATA GAT GGC AAC TCC GAG GAC GAT TGC AAA TGT CAC TGC
Y514A	F	ATC ATC GCA AGA ATC TCC ACA GCT GCA GCC TTG GCC ATG
Y514A	R	GAT TCT TGC GAT GAT AAC TCC GAG GAC GAT TGC AAA TGT
R515A	F	ATC TAT GCC ATC TCC ACA GCT GCA GCC TTG GCC ATG AAC
R515A	R	GGA GAT GGC ATA GAT GAT AAC TCC GAG GAC GAT TGC AAA
I516A	F	TAT AGA GCC TCC ACA GCT GCA GCC TTG GCC ATG AAC TCC
I516A	R	TGT GGA GGC TCT ATA GAT GAT AAC TCC GAG GAC GAT TGC
S517A	F	AGA ATC GCC ACA GCT GCA GCC TTG GCC ATG AAC TCC TCC
S517A	R	AGC TGT GGC GAT TCT ATA GAT GAT AAC TCC GAG GAC GAT
T518A	F	ATC TCC GCC GCT GCA GCC TTG GCC ATG AAC TCC TCC CCG
T518A	R	TGC AGC GGC GGA GAT TCT ATA GAT GAT AAC TCC GAG GAC
R535A	F	AAC ATC GCA GTT ACA GTC ACG GCC ACC GCT GTT ATC ATC
R535A	R	TGT AAC TGC GAT GTT GGA CCG CAC AGA CGG GGA GGA GTT
T539A	F	ACA GTC GCC GCC ACC GCT GTT ATC ATC AAC CTC GTG GTC
T539A	R	GGT GGC GGC GAC TGT AAC CCG GAT GTT GGA CCG CAC AGA
V543A	F	ACC GCT GCC ATC ATC AAC CTC GTG GTC ATC ATT CTG CTG
V543A	R	GAT GAT GGC AGC GGT GGC CGT GAC TGT AAC CCG GAT GTT
N546A	F	ATC ATC GCA CTC GTG GTC ATC ATT CTG CTG GAT GAA GTT
N546A	R	CAC GAG TGC GAT GAT AAC AGC GGT GGC CGT GAC TGT AAC
I551A	F	GTC ATC GCC CTG CTG GAT GAA GTT TAC GGC TGC ATT GCC
I551A	R	CAG CAG GGC GAT GAC CAC GAG GTT GAT GAT AAC AGC GGT
G558A	F	GTT TAC GCC TGC ATT GCC AGG TGG CTC ACC AAG ATT GAG
G558A	R	AAT GCA GGC GTA AAC TTC ATC CAG CAG AAT GAT GAC CAC
G558P	F	GTT TAC CCC TGC ATT GCC AGG TGG CTC ACC AAG ATT GAG
G558P	R	AAT GCA GGG GTA AAC TTC ATC CAG CAG AAT GAT GAC CAC
P595A	F	TAC ACT GCC ATC TTC TAT GTC GCC TTC TTC AAA GGC CGG
P595A	R	GAA GAT GGC AGT GTA AGA GTT CAC AAA CTT GAG CAG GAA
Y598A	F	ATC TTC GCC GTC GCC TTC TTC AAA GGC CGG TTT GTT GGT
Y598A	R	GGC GAC GGC GAA GAT GGG AGT GTA AGA GTT CAC AAA CTT
V599A	F	TTC TAT GCC GCC TTC TTC AAA GGC CGG TTT GTT GGT CGG

V599A	R	GAA GGC GGC ATA GAA GAT GGG AGT GTA AGA GTT CAC AAA
K603Q	F	TTC TTC CAG GGC CGG TTT GTT GGT CGG CCC GGT GAC TAC
K603Q	R	CCG GCC CTG GAA GAA GGC GAC ATA GAA GAT GGG AGT GTA
I636A	F	CTC TGT GCC CAG CTG AGC ATC ATT ATG CTG GGC AAG CAG
I636A	R	CAG CTG GGC ACA GAG CTC CAT GAG GCA GCC GCC CGG GGC
Q637A	F	TGT ATC GCC CTG AGC ATC ATT ATG CTG GGC AAG CAG CTA
Q637A	R	GCT CAG GGC GAT ACA GAG CTC CAT GAG GCA GCC GCC CGG
I640A	F	CTG AGC GCC ATT ATG CTG GGC AAG CAG CTA ATC CAG AAC
I640A	R	CAT AAT GGC GCT CAG CTG GAT ACA GAG CTC CAT GAG GCA
Q649A	F	CTA ATC GCA AAC AAT CTC TTC GAG ATT GGC ATC CCG AAG
Q649A	R	ATT GTT TGC GAT TAG CTG CTT GCC CAG CAT AAT GAT GCT

F, forward; R, reverse
