# Supplementary material for: Inhibition mechanism of the chloride channel TMEM16A by the pore blocker 1PBC
Source: Nat Commun. 2022 May 19;13:2798. doi: 10.1038/s41467-022-30479-1 (PMC9120017; doi:10.1038/s41467-022-30479-1)
Supplement: Supplementary file 3 — Reporting Summary [file 41467_2022_30479_MOESM3_ESM.pdf]

nature research

Corresponding author(s): Raimund Dutzler, Andy Lam

Last updated by author(s): Apr 11, 2022

Reporting Summary

Nature Research wishes to improve the reproducibility of the work that we publish. This form provides structure for consistency and transparency in reporting. For further information on Nature Research policies, see our Editorial Policies and the Editorial Policy Checklist.

Statistics

For all statistical analyses, confirm that the following items are present in the figure legend, table legend, main text, or Methods section.

n/a	Confirmed	
☐	☒	The exact sample size (*n*) for each experimental group/condition, given as a discrete number and unit of measurement
☐	☒	A statement on whether measurements were taken from distinct samples or whether the same sample was measured repeatedly
☐	☒	The statistical test(s) used AND whether they are one- or two-sided<br>*Only common tests should be described solely by name; describe more complex techniques in the Methods section.*
☐	☒	A description of all covariates tested
☐	☒	A description of any assumptions or corrections, such as tests of normality and adjustment for multiple comparisons
☐	☒	A full description of the statistical parameters including central tendency (e.g. means) or other basic estimates (e.g. regression coefficient) AND variation (e.g. standard deviation) or associated estimates of uncertainty (e.g. confidence intervals)
☐	☒	For null hypothesis testing, the test statistic (e.g. *F*, *t*, *r*) with confidence intervals, effect sizes, degrees of freedom and *P* value noted<br>*Give P values as exact values whenever suitable.*
☒	☐	For Bayesian analysis, information on the choice of priors and Markov chain Monte Carlo settings
☒	☐	For hierarchical and complex designs, identification of the appropriate level for tests and full reporting of outcomes
☒	☐	Estimates of effect sizes (e.g. Cohen's *d*, Pearson's *r*), indicating how they were calculated

Our web collection on statistics for biologists contains articles on many of the points above.

Software and code

Policy information about availability of computer code

Data collection	Clampex 10.7, EPU 2.9
Data analysis	Clampfit 10.7, Excel 2019, NumPy 1.20.1, SciPy 1.6.2, Pandas 1.2.4, MotionCor2 v1.4.0, Gctf 1.06, crYOLO 1.7.6, Relion 3.1.3, Phenix 1.19.2, eLBOW in Phenix 1.19.2, Coot 0.9.5, MolProbity 4.5.1, HOLE 2.2.005, charmm 39b2, UCSF Chimera 1.15, ChimeraX 1.2.5, VMD 1.9.4

For manuscripts utilizing custom algorithms or software that are central to the research but not yet described in published literature, software must be made available to editors and reviewers. We strongly encourage code deposition in a community repository (e.g. GitHub). See the Nature Research guidelines for submitting code & software for further information.

Data

Policy information about availability of data

All manuscripts must include a data availability statement. This statement should provide the following information, where applicable:

- Accession codes, unique identifiers, or web links for publicly available datasets
- A list of figures that have associated raw data
- A description of any restrictions on data availability

Data supporting the findings of this study are available from the corresponding authors upon reasonable request. The cryo-EM map, half-maps, and mask have been deposited in the Electron Microscopy Data Bank under accession number EMD-14753. Coordinates for the model are available in the Protein Data Bank under PDB 7ZK3. Protein sequences are available from UniProt: mouse TMEM16A (UniProt ID: Q8BHY3), mouse TMEM16B (UniProtID: Q8CFW1), and mouse TMEM16F (UniProt ID: Q6P9J9). Source data are provided with this paper.

April 2020

Field-specific reporting

Please select the one below that is the best fit for your research. If you are not sure, read the appropriate sections before making your selection.

☒ Life sciences ☐ Behavioural & social sciences ☐ Ecological, evolutionary & environmental sciences

For a reference copy of the document with all sections, see nature.com/documents/nr-reporting-summary-flat.pdf

Life sciences study design

All studies must disclose on these points even when the disclosure is negative.

Sample size	No sample size determination was performed. Experiments were performed multiple times with similar results and further inclusion of data did not change the results.
Data exclusions	Leaky recordings were discarded, and otherwise no data were excluded from the analyses.
Replication	Electrophysiology experiments were repeated multiple times and the number of biological replicates and errors are indicated. All attempts at replication were successful.
Randomization	Not applicable, randomization is not relevant.
Blinding	Not applicable, blinding is not relevant.

Reporting for specific materials, systems and methods

We require information from authors about some types of materials, experimental systems and methods used in many studies. Here, indicate whether each material, system or method listed is relevant to your study. If you are not sure if a list item applies to your research, read the appropriate section before selecting a response.

Materials & experimental systems

n/a	Involved in the study	
☒	☐	Antibodies
☐	☒	Eukaryotic cell lines
☒	☐	Palaeontology and archaeology
☒	☐	Animals and other organisms
☒	☐	Human research participants
☒	☐	Clinical data
☒	☐	Dual use research of concern

Methods

n/a	Involved in the study	
☒	☐	ChIP-seq
☒	☐	Flow cytometry
☒	☐	MRI-based neuroimaging

Eukaryotic cell lines

Policy information about cell lines

Cell line source(s)	HEK293T (ATCC CRL-1573) , HEK293S GnTI- (ATCC CRL-3022)
Authentication	As these are commercially available cell lines, no further cell line authentication was performed.
Mycoplasma contamination	The cell lines were tested and are free from mycoplasma contamination.
Commonly misidentified lines (See ICLAC register)	No commonly misidentified cell lines were used in the study.
